# Knowledge and psychomotor skills of nursing students in North Cyprus in the area of cardiopulmonary resuscitation

**DOI:** 10.12669/pjms.294.3450

**Published:** 2013

**Authors:** Umran Dal, Dilek Sarpkaya

**Affiliations:** 1 Umran Dal, RN, PhD, Assistant Professor, Department of Nursing, Faculty of Health Science, Near East University, North Cyprus.; 2 Dilek Sarpkaya, Research Assistant, Department of Nursing, Faculty of Health Science, Near East University, North Cyprus.

**Keywords:** Cardiopulmonary resuscitation, Basic life support, Nursing student, Nursing education

## Abstract

***Objective*** **:** The aim of the study was to determine the cardiopulmonary resuscitation (CPR) knowledge and skill levels of nursing students in North Cyprus.

***Methods*** **:** The study design was quasi-experimental and longitudinal. A questionnaire was applied to the students before the CPR lecture. Then the students were informed about adult CPR by the researchers and all of the students practiced CPR on a Resusci-Anne manikin. One and six months after this training the same questionnaire and skills checklist of CPR were applied.

***Results*** **: **Eighty three third year students of nursing participated in the study. While the average CPR knowledge score of these students was 9.3 ± 2.9 out of 23 before the lecture, this average increased to 17.0 ± 1.8 one month after the CPR lecture and decreased by two points back to 14.9 ± 3.8 after six months. Skill score of the students one month after the CPR skills training was 18.4 out of 21, and that this average decreased to 13.8 after six months (p<0.05).

***Conclusion:*** Nursing students tend to forget theoretical and applied CPR training after couple of months. Hence there is a need for continuous CPR training and education and repeating the skills at regular intervals even after they have graduated to ensure sustainability in the CPR skills.

## INTRODUCTION

According to the World Health Organization (WHO), in 2008, 17 million people (48% of all deaths) died from cardiovascular diseases, and mainly because of cardiac arrest.^1^ As per the medical statistics of North Cyprus, the primary cause of death was heart disease.^2^ Due to cardiac and respiratory arrest, many people face the risk of dying suddenly and unexpectedly and Heart conditions are one of the most common reasons for cardiac arrest.^3^^,^^4^

Cardiac arrest is a medical emergency that, in certain situations, is potentially reversible if treated early.^3^^,^^5^^-^^7^ Immediate resuscitation is necessary in order to achieve conscious survival for persons who have lost airways or pulses. Applying resuscitation procedures correctly is very important in saving the life of the individual. As nurses are generally the first healthcare professionals who realize that the patient is in cardiac arrest in a hospital, they must have an adequate level of knowledge and skills in the field of CPR which is a practice based skill.^5^^-^^10^

Nurses must improve this skill with training and repeated practice. For this reason, nurses must learn this skill, especially in their first aid lectures during nursing education. This training must be repeated, as studies have shown that the current level of CPR knowledge and skills is insufficient, and that there is a significant decrease in knowledge and skill retention of skills after a while. The training must be repeated after a specific period of time. If the skills are not used frequently, they can be forgotten over a short amount of time. CPR skills need to be maintained and practiced regularly. Repeating the CPR training will prevent the loss of knowledge and skills.^5^^-^^12^

This study was conducted with the aim of determining the level of nursing students’ knowledge and skills with regard to CPR and evaluating the effects of CPR training.

## METHODS

The study design was quasi-experimental and longitudinal. A pretest questionnaire was given before the CPR lecture in order to determine the students’ level of knowledge about the subject. The same questionnaire and a skills evaluation form were used too complete one month (posttest) and six months (retest) after the training. A total of 83 third-year students of the Department of Nursing in the Faculty of Health Sciences of Near East University in North Cyprus who were attending first aid and emergency aid lectures in the spring term of the 2009-2010 academic year participated in this study.

The data collection form was prepared in line with the literature and based on the American Heart Association’s (AHA) recommendations and International Liaison Committee on Resuscitation (ILCOR) guidelines.^4^^,^^9^^-^^13^

***Knowledge Assessment Form:*** This form was composed of 23 multiple-choice questions regarding the participants’ knowledge about CPR. In the content of the questions, there was information about ensuring that the airway was open, assessing respiration and circulation and chest compression technique. The same questionnaire was distributed before the lecture, and then one and six months afterwards. The researchers stood beside the students while these questionnaires were filled in and prevented them from helping each other. Each correct answer in the questionnaire was assigned one point, and the students were assessed with a score out of 23.

***Skills Assessment Form:*** This form was composed of 20 steps, including the steps of CPR application. These steps include skills such as finding an appropriate position in order to ensure that the airway is open, finding an appropriate position to chest compression, applying appropriate pressure and effectiveness. The psychomotor skills of the students were assessed by two researchers (One of the instructors, who conducted the research, has a master's degree in first aid and emergency aid nursing, and the other researcher has a certificate of basic life support) observing their application of CPR on the Resusci-Anne manikin in laboratory. The skills assessment form was applied one month (posttest) and six months (retest) after each student completed the applied training. The students were taken to the application laboratory by the researchers one by one and their performances using the CPR model were observed. Each correct skill on the application form was assigned one point, and the students were assessed using a score out of 20.

***Data Analysis:*** In the evaluation of the findings obtained from the study, SPSS (the Statistical Package for Social Sciences) for Windows 15.0 was used. In the study, variance analysis (ANOVA) was applied to the repeated measures and paired student's t-test was used to compare the measurements at two time points (one months and six months) after the training. The results were evaluated within the reliability range of 95% and a significance level of p<0.05.

The Institutional Review Board of Near East University approved the study. Before the study was conducted, the written permission of the Faculty Head and the verbal consent of the students were obtained.

## RESULTS

The average age of eighty three students who participated in the study was 21.2 ± 1.8 (range: 19-30) Years. Of the students, 90.4% were women and had not received any training in CPR before the study.

**Table-I:** Distribution of the correct answers provided by the students regarding CPR before and after being taught CPR

*Questions about CPR*	*Pretest* (n: 83)*		*Posttest* (one month later) n:83*		*Retest* (six months later) n: 62**	
	*N*	*%*	*N*	*%*	*N*	*%*
Definition of CPR	9	10.8	42	50.6	14	22.5
Who can perform CPR?	67	80.7	82	98.8	58	93.5
The vital minutes of CPR that it is important	46	55.4	68	81.9	46	74.2
Correct position	56	67.5	78	94.0	54	87.1
How to ensure that the airway of the unconscious patient is open if there is head and neck trauma	32	38.6	66	79.5	41	66.1
How to ensure that the airway of the unconscious patient is open if there is no head and neck trauma	31	37.3	61	73.5	36	58.1
How to start CPR	51	61.4	75	90.4	51	82.3
Whether or not thorax compression alone is sufficient	75	90.4	67	80.7	48	77.4
How to assess respiration	43	51.8	71	85.5	51	82.3
How to assess circulation	32	38.6	67	80.7	28	45.2
When to assess respiration and circulation?	14	16.9	32	38.6	25	40.3
Artificial respiration methods if there is no respiration	21	25.3	28	33.7	30	48.4
Chest compression /ventilation ratio	32	38.6	78	94.0	49	79.0
Chest compression /ventilation ratio with two rescuers (babies or children)	34	41.0	64	77.1	43	69.4
Frequency of chest compression /ventilation per minute in adults	17	20.5	46	55.4	42	67.7
The position of arms and hands during chest compression	28	33.7	62	74.7	34	54.8
Compression location in adults and children	26	31.3	39	47.0	25	40.3
Compression depth in adults	36	43.4	71	85.5	43	69.4
Thorax compression location in babies	20	24.1	36	43.4	31	50.0
Compression style in babies	23	27.7	81	97.6	52	83.9
Duration of basic life support	25	41.7	83	100.0	43	69.4
Properties of correct artificial respiration	18	21.7	69	83.1	34	54.8
Starting CPR because of sudden cardiac death and properties for calling 112	58	69.9	82	98.8	55	88.7

^*^The percentages are given based on n=83^**^The percentages are given based on n=62

As can be seen from the table Table-I, there was a significant increase in the percentage of correct answers one month after the training. Six months after the training, there was a significant decrease in the percentage of correct answers. The question about how to ensure that the airway was open was answered correctly by 37.3% of participants before the lecture, by 73.5% one month afterwards and by 58.1% six months after the lecture. The question about how to assess circulation was correctly answered by 38.6% of students before the lecture, by 80.7% one month afterwards and by 45.2% of students six months after the lecture. The number of students who knew the correct compression depth for adults was 36 (43.4%) before the lecture, 71 (85.5%) one month afterwards and 43 (69.4%) six months after the lecture.

**Table-II:** Comparison of students’ pretest, posttest and retest average knowledge scores*

	*Pretest*	*Posttest*	*Retest*	*Significance*	
	*Ave. ± (SD)*	*Ave. ± (SD)* *(one months later)*	*Ave. ± (SD)* *(six months later)*		
Change in knowledge score	9.3 ± 2.9	17.0 ± 1.8	14.9 ± 3.8	F^**^=149.624 Mauchly's W=0.863 SD=2	p= 0.000

* The increase in the average score does mean that the knowledge level increased. ** The F values in this table are Wilks’ lambda F values. As the significance level of Mauchly’s W is below 0.05, a multi-variety test was applied

As shown in the Table-II, the average knowledge score of the students before the CPR training was 9.3 out of 23 (range: 2-17). This score increased after the CPR training (17.0; range: 13-21) and decreased six months after the training (14.9; range: 6-21). The knowledge score changed significantly over time (p<0.05). In the advanced analysis which was conducted in order to determine the source of the change, it was determined that the knowledge scores in the post test and retest were significantly higher than the pretest, and that the retest scores were significantly lower than the posttest scores (p<0.05).

**Table-III:** Distribution of students’ correct skills regarding the application steps of CPR after CPR skill training

*Steps for CPR skill performance in adults*	*Posttest* (one month later) (n: 83)*		*Retest* (six months later) (n: 60)**	
	*N*	*%*	*N*	*%*
Ensuring the safety of the environment	70	84.3	44	73.3
Controlling the conscious of patient/injured, shaking them slightly, asking them “Are you feeling ok?”	75	90.4	44	73.3
Asking for medical help	71	85.5	51	85.0
Lying the patient down on a hard surface	83	100.0	60	100.0
Kneeling down beside the patient	78	94.0	55	91.7
Placing one hand on the forehead of the patient and the other one under the chin of the patient to open the airway	45	54.2	14	23.3
Tilting the head back in such a way that the mandible will be perpendicular to the floor	43	51.8	15	25.0
Determining the best spot to perform the heart massage	73	88.0	24	40.0
The correct position of the hand	80	96.4	36	60.0
Placing the other hand correctly on the hand	83	100.0	55	60.0
Interlocking the fingers of the two hands	83	100.0	55	91.7
Applying pressure on the breast bone perpendicular to the body, by not touching the chest cavity with the fingers or bending the elbows	54	65.1	34	56.7
Applying pressure in such a way that the breast bone will go down by 5 cm	74	89.2	30	50.0
30 compression , than two ventilations (30:2)	83	100.0	56	93.3
Applying the previous step 100 times per minute	71	85.5	38	63.3
Closing the nose of the patient using the thumb and index finger of the hand placed on the forehead if the patient is not breathing	68	81.9	45	75.0
Placing the mouth correctly while the patient is in a position where the head is backwards	61	73.5	22	36.7
'Two rescuers breathing for a second at a time each in a way that will help the patient’s chest to rise	76	91.6	45	75.0
Observing that the chest cavity expands when air is blown in	68	81.9	38	63.3
Continuing basic life support until the patient shows signs of life or medical help arrives	83	100.0	34	56.7

^*^The percentages are given based on n=83, ^**^The percentages are given based on n=60

In Table-III, we can see that the students’ skills of the correct CPR application steps decreased one and six months after the CPR applied training. While 54.2% of the students performed the step of ensuring the correct head/neck position for opening the airway correctly one month after the applied training, only 23.3% of the students performed this step correctly after six months. While 89.2% of the students performed the step of applying the appropriate pressure for the chest compression correctly one month after the training, only 50.0% of the students performed it correctly after six months.

**Table-IV:** Comparison of students’ posttest and retest average skill scores

	*Posttest* *(one month later)*	*Retest* *(six months later)*	*Significance*	
	*Ave. ± (SD)*	*Ave. ± (SD)*		
Change in skill score	18.4 ± 2.26	13.8 ± 3.08	t=9.639 SD=2	p= 0.000

Table-IV illustrates a comparison of the posttest and retest average skill scores of the students. It was determined that the average application score was 18.4 out of 21 one month after the CPR skill training (range: 9-21) and 13.8 after six months (range: 4-19). The skill score changed significantly over time. In the retest, the average skill score was significantly lower than in the posttest (p<0.05).

## DISCUSSION

As nurses are generally the first healthcare professionals to realize that a patient is in cardiac arrest in a hospital, they must have an adequate knowledge of CPR and the relevant skills.^5^^-^^7^. Previous studies have emphasized that CPR knowledge and skills reduce over time when they are not repeated and that the level of knowledge and skills of nurses who practice constantly is better than those who do not. ^5^^,^^9^^,^^12^^-^^14^ CPR is a skill which is based on practice. In the study that Yildirim and Celik (2008) conducted on intensive care nurses, it was determined that the knowledge scores of nurses who apply CPR frequently are higher.^14^ Studies which have revealed the need for doctors and nurses to know and apply CPR effectively have indicated very low success rates. ^5^^,^^7^^,^^10^^,^^14^^-^^16^

When we look at the results of our study, it can be seen that the number of students who correctly answered the question about the compression depth in adults was not very high before the CPR training (43.4%). However, the increase in the percentage of correct answers to this question one month after the training (85.5%) and the percentage of students who applied the correct technique (89.2%) attracted our attention. Six months later, the percentage of correct answers (69.4%) and correct applications (50%) decreased (Tables-I & III). In addition, studies have documented that the depth of the chest compressions of those who applied CPR was less successful than the other CPR steps.^9^^,^^14^^-^^16^

In a study by Yildirim and Celik. (2008), it was found that there were significant differences between the average scores that the nurses achieved on the CPR test depending on when they received CPR training^14^ The nurses’ knowledge may have lapsed after a specific period. Ensuring the sustainability of this knowledge is important for correct and reliable CPR skills. In the study by Celik (2008), the necessity of a review of CPR knowledge and skills every six to 12 months was emphasized. In the same study, it was determined that there was a significant decrease in the knowledge preservation level 10 weeks after the resuscitation course.^16^ Other studies have also determined that knowledge and skill levels decrease over time. Repeating the training periodically will prevent nurses from forgetting the knowledge and skills they have learnt.^8^^,^^10^^,^^13^^,^^16^ In our study, it was determined that the knowledge and skill levels of the nurses had decreased significantly six months after the CPR training (p<0.05).

In another study, it was found that the average scores of the nurses who had received basic life support training less than six months beforehand were higher than the average scores of the nurses who had received this training more than six months prior to the study.^14^ In hospitals, nurses are generally the health team members who see and treat the patients first. The effectiveness and timeliness of the nurses’ response is very important for keeping the patient alive until other healthcare professionals arrive. As nurses are with the patients 24 hours a day, and as they are the ones who may realize that there has been a change in one of the patients, they must have sufficient knowledge and skills in this area. An early response can save the life of a patient.

The study by Marzooq and Lyneham (2009) showed that nurses wait for their institution to offer basic life support training, instead of considering it as their responsibility to seek it out.^7^ Thus, schools and institutions have an important role in terms of planning CPR training.

***Recommendations:*** In nursing training, the theoretical and skill training should be repeated every six months, even after graduation. There should be more focus on ensuring sustainability of this training during education. The timing of nursing students training needs to be re-considered, since they are likely to require basic life support skills before they reach the third year.

## CONCLUSION

Theoretical information and practiced CPR had a positive impact on nurses’ level of CPR knowledge and practical skills in the following month. However, a significant decrease in the information preservation level and the correct application of the steps of CPR was observed six months after the training. It is important that the steps f CPR are performed correctly. 

## References

[1] World Health Organization

[2] (2011). Cyprus Medical statistics.

[3] Hardley AJ, Kaster R, Monsieurs K, Perkins G D, Davies S, Bossart L (2005). European resuscitation council guidelines for resuscitation. Adult basic life support and use of automated external defibrillators. Resuscitation.

[4] American Heart Association (AHA) American Heart Association guidelines for cardiopulmonary resuscitation and emergency cardiovascular care.

[5] Hamilton R (2005). Nurses’ knowledge and skill retention following cardiopulmonary resuscitation training: A review of the literature. J Advanced Nursing.

[6] Lima SG, Macedo LA, Vidal LM, Oliveira SA, MPB (2009). Permanent education in BLS and ACLS: Impact on the knowledge of nursing professionals. Arquivos Brasileiros de Cardiologia.

[7] Marzooq H, Lyneham J (2009). Cardiopulmonary resuscitation knowledge among nurses working in Bahrain. Intr J Nur Prac.

[8] Sener S, Guler V, Turkan H (2004). The knowledge of nurses in a training hospital about basic and advanced life support. Turkey J Emerg Med.

[9] Madden C (2006). Undergraduate nursing students’ acquisition and retention of CPR knowledge and skills. Nurse Education Today.

[10] Nicol P, Carr S, Cleary G, Celenza A (2011). Retention into internship of resuscitation skills learned in a medical student resuscitation program incorporating an immediate life support course. Resuscitation.

[11] Sener S, Yaylaci S (2010). Cardiopulmonary resuscitation and emergency cardiovascular care guideline: ‘Two guidelines and important changes to daily practice. Turkey J Emerg Med.

[12] Oermann M, Kardong-Edgren S, Odom-Maryon T (2011). Effects of monthly practice on nursing students’ CPR psychomotor skills performance. Resuscitation.

[13] Nyman J, Sihvonen M (2000). Cardiopulmonary resuscitation in nurses and nursing students. Resuscitation.

[14] Yildirim O (2008). G, Celik Oyur G. Investigation of intensive care nurses’ knowledge on basic life support. J Ege University School of Nursing..

[15] Cason CL, Kardong-Edgren S, Cazzell M, Behan D, Mancini M E (2009). Innovations in basic life support education for healthcare providers: Improving competence in cardiopulmonary resuscitation through self-directed learning. J Nurses Staff Development.

[16] Celik E (2008). The evaluation of nurses about CPR knowledge, Master's thesis.

